# Assessing vertebral bone density changes with phantomless QCT after posterior open reduction and internal fixation

**DOI:** 10.1038/s41598-025-01739-z

**Published:** 2025-05-28

**Authors:** Hu Shao, Lianda Yang, Hanfeng Hu, Ying Qian, Linlin Zhang, Huilin Yang

**Affiliations:** 1 Department of Orthopedics, The First Affiliated Hospital of Soochow University, 188#, Shizi Road, Suzhou, 215006 China; 2 Department of Radiology, The First Affiliated Hospital of Soochow University, 188#, Shizi Road, Suzhou, 215006 China

**Keywords:** Bone mineral density, Quantitative CT, Thoracolumbar fracture, Posterior open reduction and internal fixation, Trauma, Bone

## Abstract

This study was to investigate vertebral body bone mineral density (BMD) changes using a novel automatic phantom-less quantitative CT (PL-QCT) system in short and relatively long-term periods after posterior open reduction and internal fixation. 109 patients (77 men [70.6%]; median age, 48 [IQR 37, 55]) who underwent posterior open reduction and internal fixation for thoracolumbar fractures were enrolled into the study. The median follow-up duration was 441 days (IQR 377–553). Based on the median follow-up time, patients were divided into Group 1 (378 days [IQR 350–412]) and Group 2 (553 days [IQR 493–706]). Bone mineral density (BMD) was measured for the injured vertebrae, the upper and lowest instrumented vertebra, as well as the cranial and caudal adjacent vertebrae preoperatively and postoperatively. The postoperative BMD in all segments was significantly lower compared to the preoperative values (*P* < 0.05). There was no difference in BMD loss between Group1 and Group2 (*P* > 0.05). The BMD loss in the anterior region of the caudal adjacent vertebra was greater than that in the posterior region (− 13.0 mg/cm³ [IQR − 26.8, − 6.8] vs. − 12.0 mg/cm³ [IQR − 20.8, − 3.1], *P* = 0.006). Early monitoring and intervention for bone loss following surgery, particularly focusing on the anterior region of the adjacent vertebrae, could potentially aid in improving clinical outcomes with more follow-up studies.

**Supplementary Information:**

The online version contains supplementary material available at 10.1038/s41598-025-01739-z.

## Introduction

Spinal fractures are a form of orthopedic trauma that most often occurs in the thoracolumbar area. Studies have shown that thoracolumbar fractures account for approximately 90% of spinal fractures, with T11-L2 segment fractures accounting for 60–70%^1,2^. Posterior open reduction and internal fixation (ORIF) is a crucial surgical technique for treating spinal fractures. Existing literature indicates that after spinal fusion surgery, device-related osteoporosis can occur in the fused vertebrae^3^. The postoperative reduction in bone mineral density (BMD) can lead to complications such as screw loosening, adjacent segment degeneration, and recurrent fractures^4,5^. Experimental studies have shown that the reduction in BMD is associated with the stress shielding effect, biomechanical alterations, and load redistribution following the implantation of rigid spinal instrumentation^6,7^.

Dual X-ray absorptiometry (DXA) is considered the gold standard for measuring bone density. However, the accuracy of DXA measurements can be affected by spinal fixation devices, vertebral compression fractures, spinal degenerative changes and so on. Hounsfield unit values have also been used to assess bone density. A previous study has confirmed a correlation between Hounsfield units obtained from computed tomography (CT) and bone mineral density (BMD) evaluated by DXA^8^. HU values do not directly reflect bone density levels but rather serve as an indirect indicator associated with BMD. Compared to the traditional asynchronous phantom-based quantitative CT(PB-QCT) technique, a novel automatic phantom-less QCT (PL-QCT) measurement technique automatically selected regions of interest of intrinsic soft tissue (muscle or fat) to calibrate BMD measurements^9,10^. This automatic PL-QCT system offered higher accuracy than previous QCT studies while maintaining a similar diagnostic capability for osteoporosis compared to DXA and PB-QCT^9^.

Several trials have demonstrated that vertebral bone mineral density exhibited a downward trend after spinal fusion surgery. Demir et al.^11^ reported that the HU values of vertebrae in stabilized and adjacent segments consistently decreased after posterior lumbar interbody fusion. Similarly, Balci et al.^12^ found that the BMD of both the cranial and caudal adjacent vertebrae decreased after nine months following posterolateral fusion with transpedicular screw fixation. Furthermore, in patients who underwent single-segment (L4–5) transforaminal lumbar interbody fusion without drug intervention, HU values across all segments significantly decreased after 12 months^13^. However, there remains a relative scarcity of research regarding the changes in bone density of the injured vertebra, fixed vertebra, and adjacent vertebrae following ORIF for thoracolumbar fractures. Therefore, Further exploration in this area is significant for improving clinical treatment of spinal fractures, as the insights gained may inform early interventions to reduce postoperative complications like adjacent segment degeneration and recurrent fractures, thus contributing to the broader field of postoperative care and bone health.

The objective of this study was to utilize the novel PL-QCT technology to investigate the changes in BMD of the injured, instrumented, and adjacent vertebrae after posterior open reduction and internal fixation for thoracolumbar fractures. We also aimed to explore the trends of these changes, and the differences between changes in bone density in the anterior and posterior regions of the vertebrae.

## Results

We collected data from 109 cases (77 men [70.6%]; median age, 48 [IQR: 37, 55]) of thoracolumbar fractures treated with posterior open reduction and internal fixation between January 2021 and December 2023. The median follow-up duration for all patients was 441 days (IQR: 377, 553), with median follow-up times of 378 days (IQR: 350, 412) and 553 days (IQR: 493, 706) in Group 1 and Group 2, respectively. Demographic characteristics of the patients were displayed in Table 1. The distribution of vertebral fractures was as follows: 3.7% (4/109) at T12, 62.4% (68/109) at L1, 24.8% (27/109) at L2, and 9.2% (10/109) at L3.

**Table 1:** Demographic features of the surgery patients.

	All patients (*n* = 109)
Gender [male, n (%)]	77 (70.6)
Age [years, median (IQR)]	48 (37–55)
Fractured segment	
T12 [n (%)]	4 (3.7)
L1 [n (%)]	68 (62.4)
L2 [n (%)]	27 (24.8)
L3 [n (%)]	10 (9.2)
Follow-up duration [days, median (IQR)]	441 (377–553)
Genant classification	
Grade 0 [n (%)]	5 (4.6)
Grade 1 [n (%)]	32 (29.5)
Grade 2 [n (%)]	50 (45.9)
Grade 3 [n (%)]	22 (20.2)

*SD* standard deviation, *IQR* interquartile range.

Compared to preoperative vertebral BMD, the postoperative BMD at all lumbar levels was significantly reduced (Table 2). The anterior BMD of the caudal adjacent vertebrae changed by − 13.0 mg/cm³ (IQR: − 26.8, − 6.8), which was significantly greater than the posterior BMD change of approximately − 12.0 mg/cm³ (IQR: − 20.8, − 3.1) (*P* = 0.006). No significant differences in BMD loss between the anterior and posterior regions were observed in the remaining vertebrae (Table 3).

**Table 2:** Comparison of preoperative and postoperative BMD values of all segments.

	BMD1	BMD2	Z	*P* values
Cranial adjacent vertebrae (median [IQR])	153.6 (111.4-187.6)	132.4 (105.3–164.0)	− 4.586	< 0.001
Upper instrumented vertebra (median [IQR])	139.3 (113.2-177.3)	110.1 (71.1-143.7)	− 5.454	< 0.001
Injured vertebra (median [IQR])	182.3 (134.3-215.1)	153.6 (118.1-199.4)	− 3.679	< 0.001
Lowest instrumented vertebra (median [IQR])	126.9 (101.8–158.0)	110.6 (77.8-146.6)	− 5.116	< 0.001
Caudal adjacent vertebrae (median [IQR])	120.7 (94.2-155.8)	111.1 (81.6-141.4)	− 4.913	< 0.001

*BMD* bone mineral density, *IQR* interquartile range.

**Table 3:** Comparison of BMD reduction in anterior and posterior portions of vertebrae.

	Anterior vertebral ΔBMD	Posterior vertebral ΔBMD	Z	*P* values
Cranial adjacent vertebrae (median [IQR])	− 12.0 (− 34.0, − 1.9)	− 12.6 (− 38.1, 1.7)	− 0.046	0.963
Injured vertebra (median [IQR])	− 5.0 (− 54.2, 11.0)	− 5.0 (− 70.5, 10.0)	− 1.081	0.280
Caudal adjacent vertebrae (median [IQR])	− 13.0 (− 26.8, − 6.8)	− 12.0 (− 20.8, − 3.1)	− 2.769	0.006

*BMD* bone mineral density, *IQR* interquartile range.

In both Group 1 and Group 2, postoperative vertebral BMD was significantly lower than preoperative BMD (*P* < 0.05) (Tables S1, S2). There was no statistically significant difference in the BMD reduction between the two groups (*P* > 0.05) (Table 4). In Group 1, 34.5% (19/55) of patients, and in Group 2, 25.9% (14/54) of patients, showed an increase in BMD of the injured vertebra postoperatively, exceeding preoperative levels. Additionally, 25.5% of patients in Group 1 demonstrated an increase in BMD at the lowest instrumented vertebra and caudal adjacent vertebrae. Subgroup analyses revealed no statistically significant differences in BMD increase or decrease based on patient gender or age (*P* > 0.05) (Tables S3–S6).

**Table 4:** The BMD reduction of different segments according to the groups.

	Group1 (*n* = 55)	Group2 (*n* = 54)	Z	*P* values
Cranial adjacent vertebrae (median [IQR])	− 15.0 (− 50.0, − 4.0)	− 11.0 (− 23.0, − 2.5)	− 1.416	0.157
Upper instrumented vertebra (median [IQR])	− 18.3 (− 71.3, − 7.4)	− 37.4 (− 62.1, − 11.8)	− 1.282	0.200
Injured vertebra (median [IQR])	− 3.0 (− 58.7, 10.0)	− 22.6 (− 64.0, 9.9)	− 1.198	0.231
Lowest instrumented vertebra (median [IQR])	− 12.0 (− 34.6, 6.3)	− 13.0 (− 37.3, − 6.7)	− 0.826	0.409
Caudal adjacent vertebrae (median [IQR])	− 12.0 (− 15.0, 5.5)	− 12.5 (− 21.9, − 5.7)	− 0.93	0.352

*BMD* bone mineral density, *IQR* interquartile range.

## Discussion

The most commonly used techniques for bone mineral density (BMD) assessment include dual-energy X-ray absorptiometry (DXA) and quantitative computerized tomography (QCT)^14^. DXA is considered the gold standard for diagnosing osteoporosis, but it presents limitations in measuring vertebral BMD. Specifically, due to the overshadowing effect of the posterior column structures, DXA may not accurately reflect the true BMD of the vertebral body, especially in assessing changes in trabecular bone. There is a significant correlation between Hounsfield units (HU) and bone mineral density (BMD), it is important to emphasize that HU values do not directly reflect bone density levels but rather serve as an indirect indicator associated with BMD. HU values are influenced by various factors, including CT scanning techniques, equipment, parameter settings, and different types of bone tissue. Without increasing the original CT radiation dose, the post-processing of existing CT data which is called quantitative computed tomography (QCT) can yield bone density measurements that overcome the limitations of DXA in two-dimensional plane assessments. QCT is less susceptible to the degenerative changes of the spine when measuring spinal BMD^15^. In this study, we utilized a novel automatic phantom-less QCT (PL-QCT) measurement technique[9,10]. Compared to the traditional asynchronous phantom-based QCT (PB-QCT) technique, PL-QCT automatically selects intrinsic soft tissue regions, such as muscle or fat, as the region of interest (ROI) for calibrating BMD measurements. Studies have shown that the automatic PL-QCT system offers higher precision than previous QCT methods, while maintaining diagnostic accuracy for osteoporosis similar to that of DXA and PB-QCT^9^.

Several studies^11–13^ have confirmed that posterior spinal fusion surgery leaded to bone mineral density (BMD) loss in both the fixed segments and adjacent segments. However, these studies primarily focus on BMD changes following pedicle screw fixation and fusion. There is limited research on BMD changes after posterior open pedicle screw fixation without fusion for thoracolumbar fractures. Therefore, our study aimed to explore BMD changes in the injured vertebra, the upper and lower instrumented vertebrae, as well as the adjacent vertebrae following this surgical approach. Posterior open reduction and internal fixation (ORIF) was a traditional technique for treating thoracolumbar fractures. It restored the three-dimensional stability of the spine and allowed for short-segment fixation, which shortened surgery time and preserved the mobility of adjacent segments. BMD measurement was particularly important in pedicle screw fixation surgery for lumbar fractures. Preoperative BMD assessment enabled surgeons to predict the stability of screw fixation and select the most appropriate fixation strategy accordingly. Weiser et al.^16^ found a strong correlation between BMD and pedicle screw stability. When BMD was less than 80 mg/cm³, screw stability may be compromised, necessitating consideration of additional stabilization methods, such as cement augmentation. Postoperative BMD monitoring was equally crucial, as decreased BMD after pedicle screw fixation increases the risk of postoperative complications^4,5,17^, including screw loosening and fractures of adjacent vertebrae.

In this study, the median follow-up duration was 441 days (IQR: 377–553). Compared to preoperative values, we observed a significant postoperative decrease in BMD across the injured vertebra, the upper and lower instrumented vertebrae, and adjacent vertebrae (*P* < 0.001). These findings were consistent with previous studies^11^, which reported a continuous decrease in HU values of both stabilized and adjacent segments 9 to 13 months after lumbar pedicle screw fixation and fusion surgery for lumbar degeneration and spinal stenosis. According to Wolff’s law, bone was a dynamic structure that constantly remodeled in response to mechanical forces. Regular stress on bones promoted bone formation and maintained bone density. However, the pedicle screw fixation system, which provided strong stabilization of the three columns of the spine, bore most of the load. This reduced the load on the stabilized segments, leading to stress shielding, which caused issues such as osteoporosis, bone atrophy in the stabilized segments, adjacent segment degeneration, and hardware failure like screw or rod breakage due to concentrated stress. Our findings confirmed that significant BMD reductions occurred in the injured vertebrae, instrumented vertebrae, and adjacent vertebrae following pedicle screw fixation. To create an ideal mechanical environment for the stabilized and adjacent segments, proper stress stimuli were essential, implying that spinal fixation should aim to reduce rigidity and enhance load sharing, without compromising stability.

When comparing the anterior and posterior BMD changes of the vertebral bodies, we found that in the lower adjacent vertebra, the anterior BMD loss was greater than that of the posterior region. No significant differences were found in the other segments. This discrepancy may be attributed to biomechanical changes following the implantation of the screw-rod system^6,7^. After the implantation, the distribution of load across the vertebral bodies was altered, with the anterior region potentially experiencing greater mechanical stress, which leads to increased bone resorption in that area. Meanwhile, the posterior structures may bear less load due to the stabilization provided by the fixation system. This redistribution of forces, along with changes in spinal kinematics, means that the anterior portion of adjacent vertebrae was more susceptible to bone density loss, particularly in cases where pre-existing conditions such as osteoporosis were present.

To further investigate the trend of BMD reduction, we divided the 109 patients into two groups based on the median follow-up time: Group 1 (378 days [IQR: 350–412]) and Group 2 (553 days [IQR: 493–706]). In both groups, all vertebral segments showed significant reductions in BMD compared to preoperative values, but there was no significant difference in the BMD changes between the two groups (*P* > 0.05). This suggested that BMD decreases gradually during early postoperative follow-up and then stabilized. Zhang et al.^18^ observed similar findings in 86 patients who underwent short-segment lumbar fixation, where adjacent vertebrae showed varying degrees of BMD reduction at 3, 6, 12, and 18 months, with the most significant reduction occurring at 12 months, followed by a slight increase at 18 months. Similarly, Balci et al.^12^ reported BMD reductions in both cranial and caudal adjacent vertebrae during an average follow-up of 9 months after lumbar surgery, with this loss persisting after an average follow-up of 32 months but without worsening. No statistically significant difference was found between the BMD changes at 9 and 32 months based on preoperative values (− 12.3 ± 16.1 mg/cm^3^ vs. −13.2 ± 21.5 mg/cm^3^, *P* > 0.05). Following fractures, mechanical unloading, inflammation, and hormonal changes regulating calcium homeostasis led to greater osteoclast activity than osteoblast activity, resulting in bone loss. Over time, the body’s self-regulation and adaptation activate osteoblastic effects, and as the balance between osteoblastic and osteoclastic activities is restored, BMD reaches a plateau. Therefore, early monitoring and intervention for bone loss following fracture surgery are essential to promote fracture healing and reduce the risk of secondary fractures.

In Group 1 and Group 2, 34.5% (19/55) and 25.9% (14/54) of patients, respectively, showed an increase in BMD at the injured vertebra, even surpassing preoperative levels. According to subgroup analysis, there was no statistically significant difference in BMD changes by gender or age. In Group 1, 25.5% (14/55) of patients showed an increase in BMD in the lower instrumented vertebra and lower adjacent vertebra, even exceeding preoperative levels, but in Group 2, BMD remained lower than preoperative levels in all patients.

This study primarily explores BMD changes in various vertebral segments after posterior open reduction and internal fixation. However, there are several limitations to this study. First, as a retrospective study, there were inherent biases, and the number of included patients was limited. The conclusions of this study required validation in larger, prospective trials. Second, Although the enrolled patients all underwent single-segment fixation, we did not control for the specific segments that were fixed. Different fixed segments may endure varying biomechanical loads, which could influence changes in bone density and recovery. Third, the measurement of BMD in fractured and instrumented vertebrae presents a challenge. Although we categorized fracture severity using the Genant classification and primarily included patients with mild to moderate fractures (Genant Grades 0–2), in some severe cases (Genant Grades 2–3), it was difficult to completely exclude regions with high-density areas caused by fracture-related compression. This may have resulted in an overestimation of BMD in certain instances. Fourth, we only analyzed the effects of age and gender on BMD changes. Future studies should consider additional factors, such as BMI, hormone levels, and the influence of medications.

## Methods

### Materials and participants

This was a retrospective study aimed at investigating the changes in vertebral bone mineral density (BMD) in patients who underwent posterior open reduction and internal fixation (ORIF) for thoracolumbar fractures between January 2021 and December 2023. The inclusion criteria were as follows: (1) patients aged > 18 years; (2) patients with fresh single-segment thoracolumbar fractures; (3) patients who received posterior open reduction and internal fixation; (4) patients who underwent CT examinations both preoperatively and postoperatively. The exclusion criteria included: (1) history of old vertebral compression fractures, severe multiple injuries, or fractures at other sites; (2) vertebral tumors or tuberculosis; (3) previous history of thoracolumbar surgery; (4) failure to follow up. The study was approved by the Ethics Committee of the First Affiliated Hospital of Soochow University, Soochow, China. This retrospective study was approved by the Ethics Committee of the First Affiliated Hospital of Soochow University, which waived the requirement for obtaining informed consent from participants due to the retrospective nature of the study. All methods were carried out in accordance with relevant guidelines and regulations.

The interval between the two CT examinations in this study was 441 days (IQR: 377–553). Based on the median interval, patients were subsequently divided into two groups. Group 1 (interval ≤ 441 days) had a median follow-up time of 378 days (IQR: 350–412), while Group 2 (interval > 441 days) had a median follow-up time of 553 days (IQR: 493–706).

### Surgical procedures

The patient was positioned in the prone position, with the target intervertebral space supported by a pad. Preoperative fluoroscopy using a C-arm was employed for localization. An electric scalpel was used to incise the skin and subcutaneous tissue, followed by muscle dissection using a periosteal elevator to expose one spinal segment above and below the fracture site. A rongeur was used to prepare the entry point for the insertion of a Kirschner wire. After confirming the position under C-arm fluoroscopy, a standard pedicle screw was implanted. Fluoroscopy was repeated to verify the screw’s position and length. A rod holder was then used to insert a titanium rod of appropriate length and curvature, which was secured by tightening the nuts. Finally, a transverse connector was inserted and locked until the titanium rod was fully secured.

### CT acquisition and assessment of BMD

All patients underwent preoperative and postoperative non-contrast CT scanning centered on the injured vertebral body using a 256-slice spiral CT scanner (manufacturer: Philips, model: Brilliance). The patients were placed in a supine position. The scanning parameters included a tube voltage of 120 kV, a tube current of 185 mAs, a field of view 512 mm × 512 mm, a slice thickness of 0.6 mm, a slice interval of 0.6 mm, a window width of 1450 HU, and a window level of 450 HU.

**Fig. 1:**
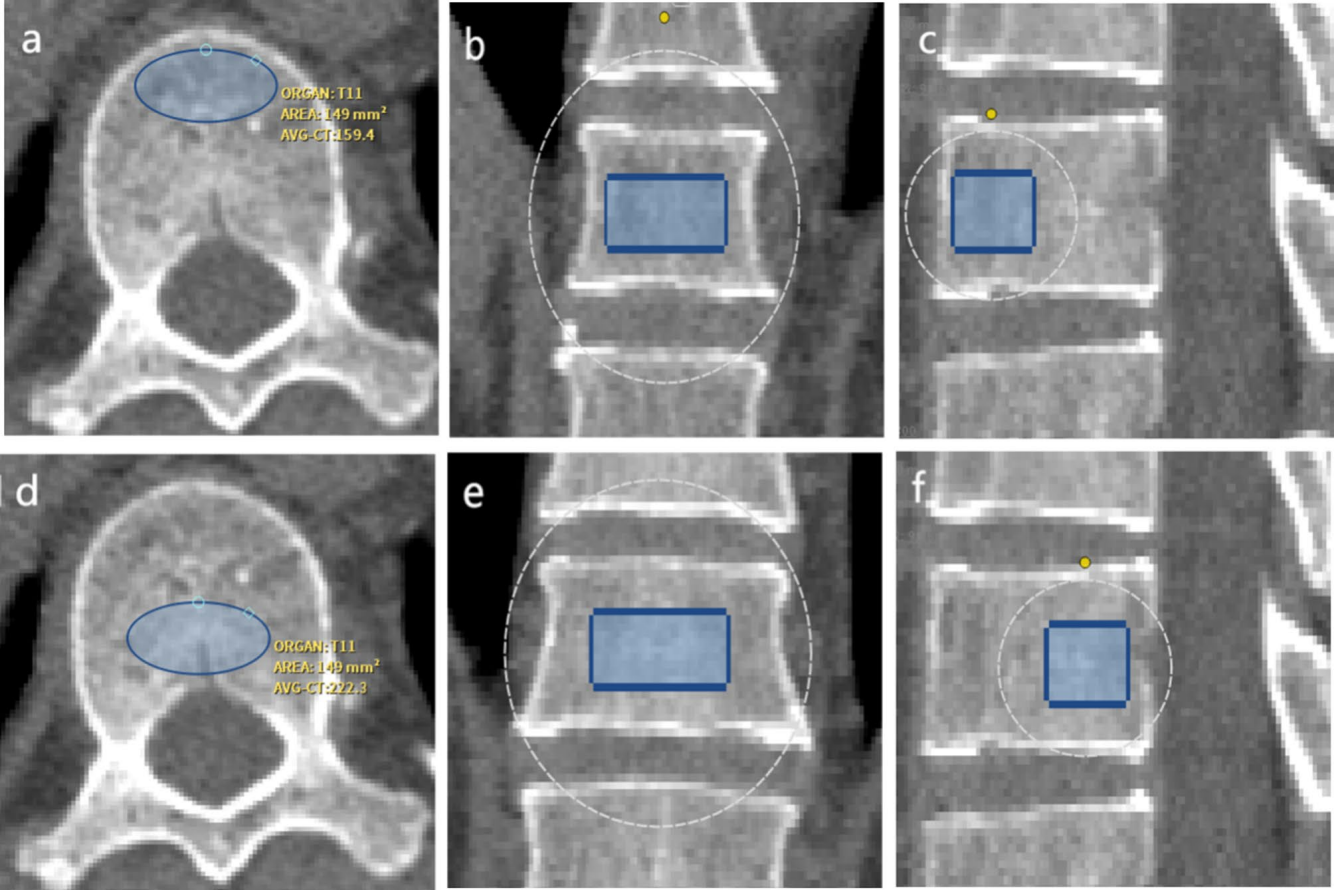
CT images showing bone density measurement assessments of the anterior and posterior vertebral body in axial, coronal, and sagittal planes. Figures (**a–c**) present the axial, coronal, and sagittal measurements of bone density in the anterior vertebral body. Figures (**d–f**) display the axial, coronal, and sagittal measurements of bone density in the posterior vertebral body.

**Fig. 2:**
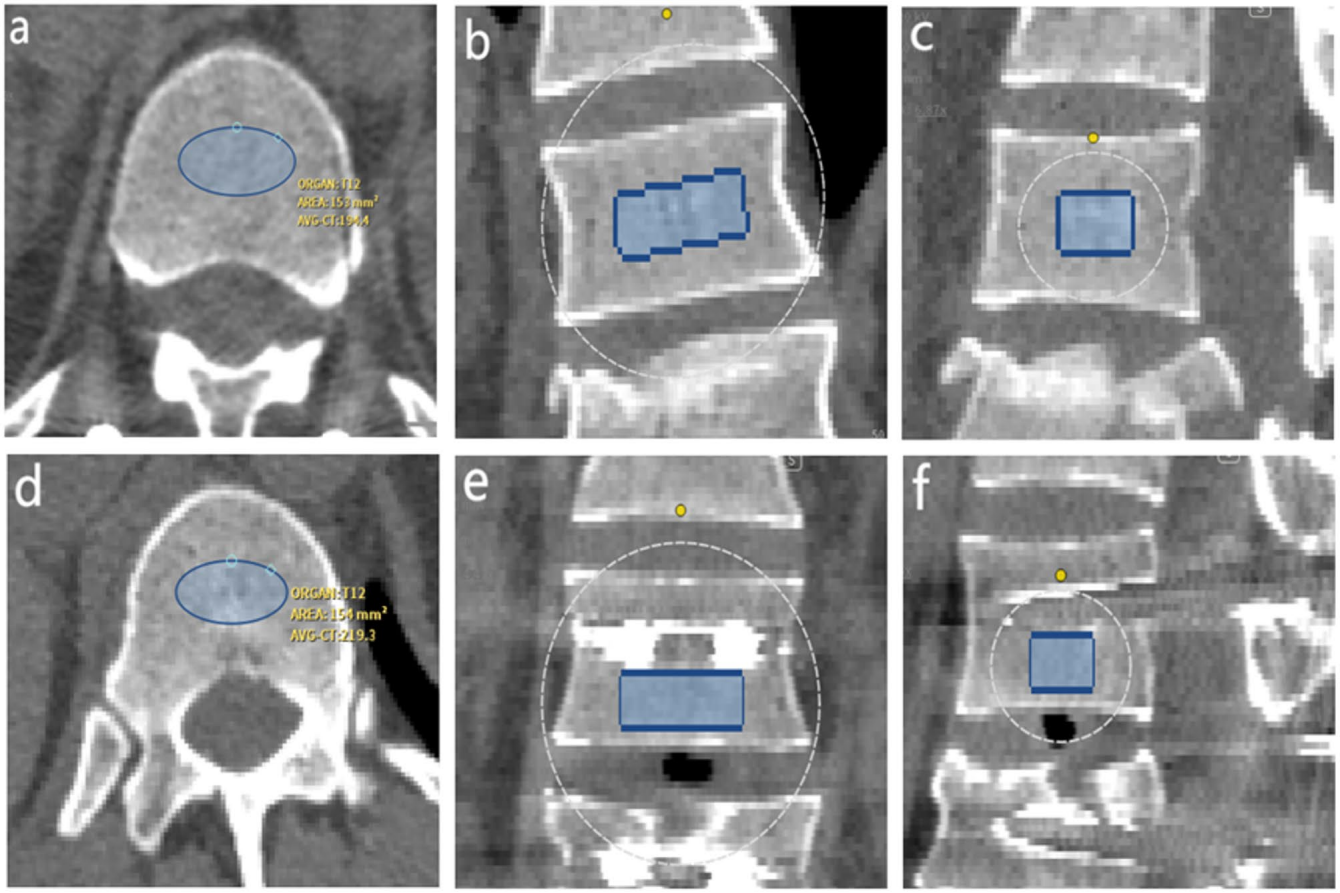
CT images showing bone density measurement assessments of the preoperative and postoperative vertebral body with pedicle screws in axial, coronal, and sagittal planes. Figures (**a–c**) present the axial, coronal, and sagittal measurements of bone density in the preoperative vertebral body, while Figures (**d–f**) display the axial, coronal, and sagittal measurements of bone density in the postoperative vertebral body.

Automatic phantom-less quantitative CT (QCT) (Bones QCT, Bones Technology Limited, Hong Kong, China)^9,10^ was employed to measure volume bone mineral density. The new technique used subcutaneous fat and paraspinal muscles as internal references. The coordinates of the regions of interest (ROI) of the internal soft tissue were automatically placed for bone mineral density (BMD) calibration. The ROI of vertebrae was an ellipsoid placed centrally within the vertebral body without contacting the cortical bone. The ROI area was approximately 150 mm². For the cranial and caudal adjacent vertebrae, the measurement height was set at 9 mm. For the injured vertebra and instrumented vertebrae, the measurement height was 6 mm to avoid interference from pedicle screws. Additionally, before performing the measurements, we applied a metal artifact reduction process to minimise the influence of pedicle screws, ensuring more accurate BMD assessment. During the measurement, we adjusted the ROI to ensure that its top and bottom edges were aligned parallel to the inferior endplate of the vertebra. The preoperative and postoperative BMD of the injured vertebra, upper and lowest instrumented vertebra, as well as cranial and caudal adjacent vertebrae were measured. Anterior vertebral BMD was defined as the BMD of the anterior half of the vertebral body, while posterior vertebral BMD was defined as the BMD of the posterior half of the vertebral body. Figure 1 showed the measurement of the anterior and posterior half of the vertebral body and the preoperative and postoperative measurements were displayed in Fig. 2. The preoperative BMD of the target vertebra was recorded as BMD1, and the postoperative BMD at follow-up was recorded as BMD2. The change in BMD (ΔBMD) was calculated as ΔBMD = BMD2 − BMD1.

### Statistical analysis

The Shapiro-Wilk test was employed to assess the normality of all continuous variables. Continuous variables following a normal distribution were presented as mean ± standard deviation (Mean ± SD) and compared using the paired t-test. Otherwise, continuous variables were expressed as the median and interquartile range (Median [IQR]) and compared using the Wilcoxon signed-rank test. Categorical variables were reported as frequency and percentage (N, %). For subgroup analyses, continuous variables with normal distribution were compared using the independent samples t-test; otherwise, the Mann-Whitney U test was applied for non-normally distributed data. Categorical variables were compared using the Chi-square test. Two-sided *P*-values of less than 0.05 were considered statistically significant. The statistical analysis was carried out using the Statistical Package for the Social Sciences (SPSS) version 23.0.

## Conclusions

Significant reductions in bone mineral density (BMD) were observed in all vertebral segments after posterior open reduction and pedicle screw fixation for thoracolumbar fractures. Differences in bone loss between the anterior and posterior regions of the vertebral body may be linked to biomechanical changes from the screw-rod system implantation, necessitating further investigation. While BMD decreased during early postoperative follow-up, no further deterioration was noted in the longer-term follow-up. Thus, monitoring and early intervention for post-surgical bone loss may help enhance clinical outcomes with additional follow-up studies required to validate this approach.

## Electronic supplementary material

Below is the link to the electronic supplementary material.

Supplementary Material 1

## Data Availability

The datasets generated and analyzed during the current study are not publicly available because they contain private patient health information but are available from the corresponding author on reasonable request.
