# Supplementary material for: Assessing vertebral bone density changes with phantomless QCT after posterior open reduction and internal fixation
Source: Sci Rep. 2025 May 28;15:18665. doi: 10.1038/s41598-025-01739-z (PMC12119896; doi:10.1038/s41598-025-01739-z)
Supplement: Supplementary file 1 — Supplementary Material 1 [file 41598_2025_1739_MOESM1_ESM.pdf]

Supplementary material

Table S1. Comparison of preoperative and postoperative BMD values in Group1

	BMD1	BMD2	Z	*P*
Cranial adjacent vertebrae (Median [IQR])	155.3(122-195.9)	136.3(106.3-164.0)	-3.503	<0.001
upper instrumented vertebra (Median [IQR])	139.3(117.8-177.5)	118.5(70.8-158.6)	-2.807	0.005
injured vertebra (Median [IQR])	176.8(127.6-209.6)	146.9(118.7-191.9)	-2.130	0.033
lowest instrumented vertebra (Median [IQR])	129.4(104.4-161.5)	123.4(77.7-156.6)	-2.600	0.009
Caudal adjacent vertebrae (Median [IQR])	126.7(95-158.6)	113.7(82.7-149.5)	-2.514	0.012

BMD, bone mineral density; IQR, interquartile range

Table S2. Comparison of preoperative and postoperative BMD values in Group2

	BMD1	BMD2	Z	*P*
Cranial adjacent vertebrae (Median [IQR])	141.25(106.8-180.45)	132.15(102.525-166.5)	-3.010	0.003
upper instrumented vertebra (Median [IQR])	139.75(103.875-176.225)	98.85(71.075-132.4)	-5.072	<0.001
injured vertebra (Median [IQR])	186.1(142.65-225.375)	156.05(113.125-204.5)	-2.894	0.004
lowest instrumented vertebra (Median [IQR])	124.45(96.225-155.95)	109.7(77.775-134.075)	-4.724	<0.001
Caudal adjacent vertebrae (Median [IQR])	112.15(90.575-148.925)	101.4(74.225-134.575)	-4.485	<0.001

BMD, bone mineral density; IQR, interquartile range

Table S3. Age- and gender-based subgroup analysis of injured vertebral bone changes in Group1

	ΔBMD >0 (n=19)	ΔBMD <0 (n=36)	*P*
Age [years, (Mean ± SD)]	48.79±3.822	45.19±2.067	0.369
Gender			0.282
Male[n (%)]	11(57.9)	26(72.2)	
Female [n (%)]	8(42.1)	10(27.8)	

BMD, bone mineral density; SD, standard deviation;

Table S4. Age- and gender-based subgroup analysis of injured vertebral bone changes in Group2

	ΔBMD >0 (n=14)	ΔBMD <0 (n=40)	*P*
Age [years, (Mean ± SD)]	43.57±2.682	47.41±2.352	0.492
Gender			0.927
Male[n (%)]	11(78.6)	29(72.5)	
Female [n (%)]	3(21.4)	11(27.5)	

BMD, bone mineral density; SD, standard deviation;

Table S5. Age- and gender-based subgroup analysis of lowest instrumented vertebral bone changes in Group1

	ΔBMD >0 (n=14)	ΔBMD <0 (n=41)	*P*
Age [years, (Mean ± SD)]	48.79±3.822	45.19±2.067	0.379
Gender			0.206
Male[n (%)]	7(50.0)	30(73.2)	
Female [n (%)]	7(50.0)	11(26.8)	

BMD, bone mineral density; SD, standard deviation;

Table S6. Age- and gender-based subgroup analysis of Caudal adjacent vertebral bone changes in Group1

	ΔBMD >0 (n=14)	ΔBMD <0 (n=41)	*P*
Age [years, (Mean ± SD)]	44.43±2.857	47.12±2.340	0.538
Gender			0.545
Male[n (%)]	8(57.1)	29(70.7)	
Female [n (%)]	6(42.9)	12(29.3)	

BMD, bone mineral density; SD, standard deviation;
